# Pleistocene glaciations, demographic expansion and subsequent isolation promoted morphological heterogeneity: A phylogeographic study of the alpine *Rosa sericea* complex (Rosaceae)

**DOI:** 10.1038/srep11698

**Published:** 2015-06-30

**Authors:** Yun-Dong Gao, Yu Zhang, Xin-Fen Gao, Zhang-Ming Zhu

**Affiliations:** 1 Key Laboratory of Mountain Ecological Restoration and Bioresource Utilization, Chengdu Institute of Biology, Chinese Academy of Sciences.

## Abstract

While most temperate plants probably underwent glacial constriction to refugia and interglacial expansion, another type of interglacial refugia might have existed to maintain alpine plants during warm periods. To test this hypothesis, we applied phylogeographic methods to 763 individuals (62 populations) which belong to 7 taxonomically difficult species of the *Rosa sericea* complex distributed in alpine regions of the temperate and subtropical zones in eastern Asia. We used three chloroplast (cp) DNA fragments (*trn*L-*trn*F, *ndh*F-*rpl*32 and *ndh*J*-trn*F) approximately 3,100 bp and nuclear microsatellite (nSSR) on eight sites to determine whether cold tolerant plants experienced expansion during the Pleistocene. The neutral test and mismatch distribution analysis (MDA) indicated that whole populations and major lineages of the Qinghai-Tibet Plateau (QTP) underwent expansion during the middle to late Pleistocene. Environmental niche modeling (ENM) indicates more suitable habitats during the Last Glacial Maximum (LGM) than at present. We concluded that the demographic history of *R. sericea*, which diverged in the middle Pleistocene, was mostly affected by climatic oscillations instead of by geographical barriers. The low genetic divergence, as well as the weak phylogenetic structure in the *R. sericea* complex both support treating this complex as a single taxon.

Paleoclimatic and paleogeological events have been identified as being the two major drivers of genetic variation in extant organisms. Geographic changes, such as the uplifting of the Qinghai-Tibet Plateau and Rocky mountain systems, result in habitat fragmentation that forms barriers to gene flow, further leading to genetic diversification, accelerated variation and even speciation^1^,^2^,^3^,^4^,^5^. On the other hand, climatic oscillations during the Quaternary also played a significant role in affecting the demographic history of many temperate plants, which experienced glacial retreat and postglacial expansion in their ranges (e.g., Qiu *et al.*^6^). Repeated glacial retreats and postglacial expansions of the ranges of many plants profoundly shaped their distribution patterns in eastern Asia and in many other regions of the Northern Hemisphere^7^,^8^. In most cases, plants that were not cold tolerant (temperate species) were restricted to relatively warmer places during glacial cycles where they occurred in shelters, now identified as refugia^8^. However, the effects of Quaternary climatic oscillations on the population dynamics of cold tolerant plants is not yet clear, especially for those plants in temperate zones.

Two theories, named the pioneer model and the phalanx model, have been advanced to address the consequences of population expansion on genetic legacy^7^,^8^. The ‘pioneer’ model proposed by Hewitt^7^ predicts that a small pioneer or edge populations expand from a refugium and should exhibit low genetic diversity as a result of the founder effect. Not entirely in contrast, the ‘phalanx’ model of spread^7^ predicts that large populations with pronounced genetic structure expand slowly from refugia and that sharp contact zones may occur at colonizer fronts. Compared to the pioneer model, which is well-documented, the phalanx model has received less attention despite its potential importance in the demographic history of montane (especially alpine) species. In particular, The phylogeographic structure of high elevation species may have been shaped by several cycles of interglacial allopatric fragmentation followed by glacial contiguous range expansion consistent with the phalanx hypothesis along an elevational gradient^9^,^10^,^11^,^12^. Compared to the former model that was well documented, the phalanx model was less consulted despite its potential importance in the demographic history of montane (especially alpine) species.

In recent years, phylogeographic research has shown that glaciation may not only force population contractions in plants, but also promote expansion or persistence of cold adapted or cold tolerant species. For example, studies in *Dryas integrifolia*^13^; *Picea glauca*^14^; *Pinus kwangtungensis*^15^; juniper^16^, and animals, such as lemmings^17^,^18^, rock ptarmigan (*Lagopus muta)*^19^, mountain sheep^20^ and Aura^21^ have shown cold adaption and demographic expansion during cold periods. Therefore, it seems likely that glacial expansions and interglacial contractions were a recurrent pattern for cold adapted species during the Late Quaternary.

Although recent phylogeographic studies have been conducted on various plant groups and many geographic regions^21^,^22^,^23^,^24^,^25^, few have concentrated on taxonomically complex groups. Phylogeographic studies on monophyletic groups, especially taxonomically difficult groups, may provide a comprehensive understanding of demographic history, potential interspecific gene flow and species delimitation in long controversial taxa. On the other hand, incorrect phylogeographic inference could be avoided with a more comprehensive sampling strategy. In taxonomically difficult taxa, focusing on a single species was not feasible. Instead, a monophyletic group should be favored^26^.

The *Rosa sericea* complex comprises a group of shrubs in section *Pimpinellifoliae* DC. Ser. *Sericeae* (Crep.) Yu et Ku^27^,^28^ of the family Rosaceae. The flowers have white or yellowish petals and four tepals. The complex represents a typical alpine shrub with a distribution in the Sino-Himalayan region extending eastward into low elevation montane areas of western Guizhou and Hubei provinces at elevations between 1,400 and 4,500 m. With deceasing elevation, the complex is absent in southeastern China, but with one taxon disjunctly distributed at high elevations in the central mountains of Taiwan (Fig. 1). According to recent phylogenetic studies of our research team^29^, the *R. sericea* complex is a monophyletic group most closely related to *R. hugonis* Hemsl. and contains seven species: *R. sericea*, *R. omeiensis*, *R. zhongdianensis*, *R. sikangensis*, *R. morrisonensis*, *R. taronensis*, *R. mairei* or infraspecific taxa^30^. Compared to other shrubs endemic to QTP, the *R. sericea* complex has a broader eastward distribution. The complex may have had a continuous distribution in southeastern China in the past, and is a good candidate for a demographic study of cold adapted species.

In this study, we used DNA sequences and a phylogeographic approach as well as niche modeling to determine the mechanism of range expansion and disjunction within the *R. sericea* complex. In particular, we wanted to answer these questions: 1) Did range expansion exist in the demographic history of *R. sericea*? If it did, which factors (e.g., interglaciations, glaciations or others) contributed to this expansion? 2) How were southwestern China – Taiwan disjunctions formed? Did continuous distributions once exist in eastern China? If so, then which part served as a bridge between the two areas? In addition, we also aimed to determine suitable species delimitations for the *R. sericea* complex.

## Results

### Sequence characteristics and within-population genetic diversity

The three cpDNA-IGS surveyed across 691 individuals (belonging to 62 populations) were aligned. Of the 3057 bp, nucleotide substitutions occurred at 32 sites and 10 indels (from 1 to 24 bp) were present (Table S3). After indels were reduced and treated as single mutation events, the aligned length was 2972 bp, and contained 42 variable sites of which 32 sites were parsimony informative. A total of 37 chlorotypes were identified according to these combination polymophisms. Table 1 shows basic genetic data and statistics for all populations in the regions that we studied.

**Figure 1:**
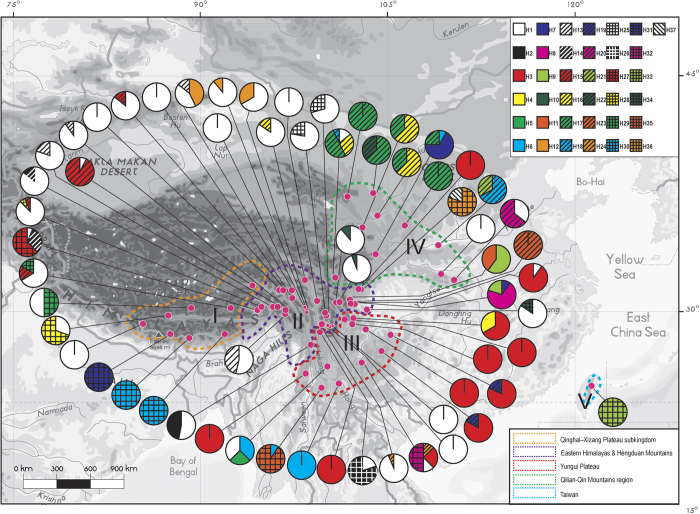
Geographical distribution of 37 chlorotypes (H1-H37) identified in 62 populations of *Rosa sericea* complex. Dashed lines delimit five population groups (I-V) divided by geographic features by Wu *et al.*^54^. The map generated by ESRI ArcGIS 9.3 (ERSI co., USA).

Total nucleotide (*π*) and haplotype (*H*_d_) diversity across all populations was 0.00056 and 0.815. The QTP group exhibited the highest haplotype diversity (*H*_d_ = 0.779) while the largest group in Hengduan Mountains (region II in Fig. 1) group possessed the lowest (*H*_d_ = 0.522). Genetic diversity calculated for all populations (*H*_T_ = 0.823) was higher than the average intra-population diversity (*H*_S_ = 0.267). Relative high levels of genetic differentiation were observed (*G*_ST_ = 0.676, *N*_ST_ = 0.766). Permutation tests showed that *N*_ST_ was greater than *G*_ST_ in all groups and in the metapopulation (Table 2), suggesting the existence of phylogeographic structure (P < 0.01, Table 2).

### Nuclear microsatellite genotyping

The 62 populations of the *Rosa sericea* complex (n = 595) revealed a total of 254 alleles across the eight nSSR loci surveyed, along with high mean per-locus estimates of allele and gene diversity (N_A_ = 24.4; H_S_ = 0.567; H_T_ = 0.807; see Table S2). Population differentiation (F_ST_) was significant at each locus (P< 0.05) and averaged 0.298 across all loci (Table S2).

**Figure 2:**
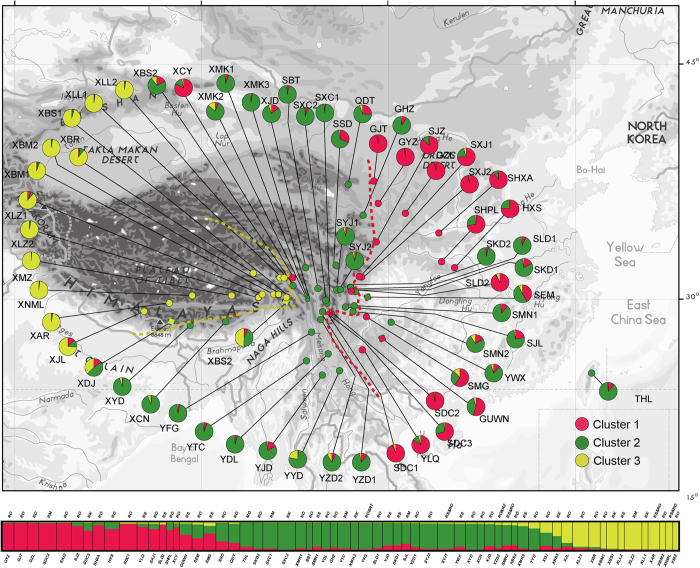
Geographic origin of the 62 *R.sericea* complex populations and their colour-coded grouping according to the STRUCTURE analysis. Population codes are identified in Table 1. The bottom showed a histogram of the STRUCTURE assignment test for 62 populations (763 individuals) of *R. sericea* complex based on variation at eight nuclear microsatellite (nSSR) loci. Population codes and abbreviation for species are identified in Table 1. The map generated by ESRI ArcGIS 9.3 (ERSI co., USA).

For the entire nSSR dataset (62 populations, n = 763), STRUCTURE yielded the highest likelihood when samples were clustered into three groups (K = 3, data not shown). The pre-defined taxa and the recognized clusters in *R. sericea* complex showed no relativeness, but distinct geographic patterns can be detected (Fig. 2). Three mainly regions were recognized as Tibetan Plateau, Hengduan Mountains and eastern edge of QTP (divided by lines in Fig. 2). Contact among populations in the three regions was detected as heterozygosity (Fig. 2).

### Population genetic and phylogeographic structure

Spatial genetic analyses of cpDNA haplotypes in 62 populations using SAMOVA indicated that *F*_CT_ increased with partitioning of species into 2 to 20 groups (Fig. S1). When the 62 populations were evenly subdivided into 20 groups, there was still increase variation among divided groups. Thus for AMOVA analysis, we divided the whole sampling area into five groups mainly based on geographic features (Table 1, Fig. 1). The results showed that 20.76% of the total vicariance was distributed within populations (*F*_ST_ = 0.822, p < 0.0001), and a large level of variation was due to differentiations between populations within geographic regions (56.82%, *F*_SC_ = 0.761, p < 0.0001) and among geographical regions (PV = 25.33%, *F*_CT_ = 0.253. P < 0.0001) (Table 3). The AMOVA analysis of groups of separated taxa showed a very low level of variation among the seven species traditionally recognized according to morphology (PV = 7.36%, *F*_CT_ = 0.074, P < 0.05) (Table S4).

### Phylogenetic relationships of cpDNA haplotypes and divergence time estimates

**Figure 3:**
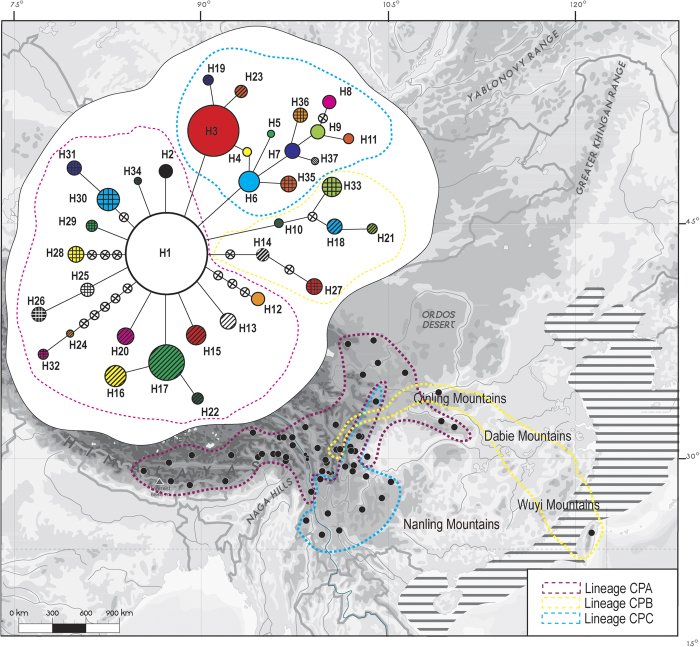
A network of cpDNA haplotypes constructed by TCS 1.21. Size of circles in network are proportional to observed frequencies of chlorotypes. Three lineages were identified as referencing the phylogenetic result in Fig. S2. The background dots showed the sample locations and gross distribution of these three lineages (CPA-CPC). Shade part indicated the LGM coastline extension which is −130 m compared to present. The map generated by ESRI ArcGIS 9.3 (ERSI co., USA).

The tree topologies generated by Neighbor-joining (NJ) and Bayesian inference (BI) were congruent in terms of major clades, thus only the NJ tree is presented in Figure S2. Overall, 37 chlorotypes were clustered into three major lineages: 1) northern Hengduan Mountains and QTP, 2) southern Hengduan Mountains and Yungui Plateau, and 3) eastern China, named CPA to CPC, respectively (Fig. S2). This was congruent with the parsimony network, which showed the 37 chlorotypes grouped into three major clades separated by only one mutational step (Fig. 3). We separated the three haplotype lineages for demographic analysis. The separation appears to be artificial since they did not receive strong support, although they demonstrated basic geographic information in such context. As the whole network showed, the *Rosa sericea* complex presented a radiation-shaped relationship, with a weak phylogeographic pattern (Fig. 3). We separated all chlorotypes into three lineages with the aim to obtain the maximum amount of information on demographic history.

Assuming substitution rates of 2.0 × 10^−9^ s/s/y, the divergence time for the *Rosa sericea* complex was estimated to be 0.482 Ma, with a 95% confidence interval (95% CI) of 0.281–0.722 Ma (Fig. S1). We used lower and upper bounds on the rates of nucleotide substitution of 1.1 × 10^−9^ s/s/y and 2.9×10^−9^ s/s/y, respectively, and estimated that the divergence time within this complex was 0.935 (95% CI 0.480–1.269 Ma) and 0.352 Ma (95% CI 0.175–0.469 Ma), also espectively. Overall, all our results indicate a mid-Pleistocene divergence of populations within this complex.

### Demographic history

Since SAMOVA did not provide optimal K to define the group (Fig. S1), we used the lineages identified by phylogeny and network (see results above) for mismatch distribution analysis (MDA). We applied MDA analysis to the three lineages CPA-CPC and the entire chlorotypes of the *Rosa sericea* complex using a sudden demographic expansion model. The results showed that the whole populations and CPA lineage were unimodal, while lineages CPB and CPC were bimodal and multimodal, respectively. Accordingly, the two statistics –*SSD* and *H*_Rag_ – that assessed the fitness of the model only rejected the expansion model in lineage CPC. In neutrality tests, the whole group, lineage CPA and CPB showed negative values in Tajima’s *D* and Fu’s *F*s test, which indicated population expansion. Only the whole lineage, however, showed significant negative value for Fu’s *F*s (p < 0.0001, Table 4). The neutrality tests both supported a demographic expansion in the past in the CPA lineage, although not significantly (0.01 < p < 0.05, Table 4). This was consistent with the MDA results that indicated both of these two sets were unimodal while the rest were not. Despite the good fitness of the expansion model to lineage CPB with bimodal distributions in MDA, this is not taken as strong evidence for expansion at present, because multimodal distributions generally indicate either populations that are stable or shrinking in size or structure^31^,^32^. Thus, based on the corresponding *τ* value, and assuming minimum and maximum mutation rates of 1.1 × 10^−9^ s/s/y and 2.9 × 10^−9^ s/s/y, the expanding event for the whole group was dated as 425 k years and 112 k years BP, respectively (Table 4). Bayesian skyline plots suggested that effective population sizes increased slowly during the LGM (Fig. S4).

### Palaeodistribution modeling

**Figure 4:**
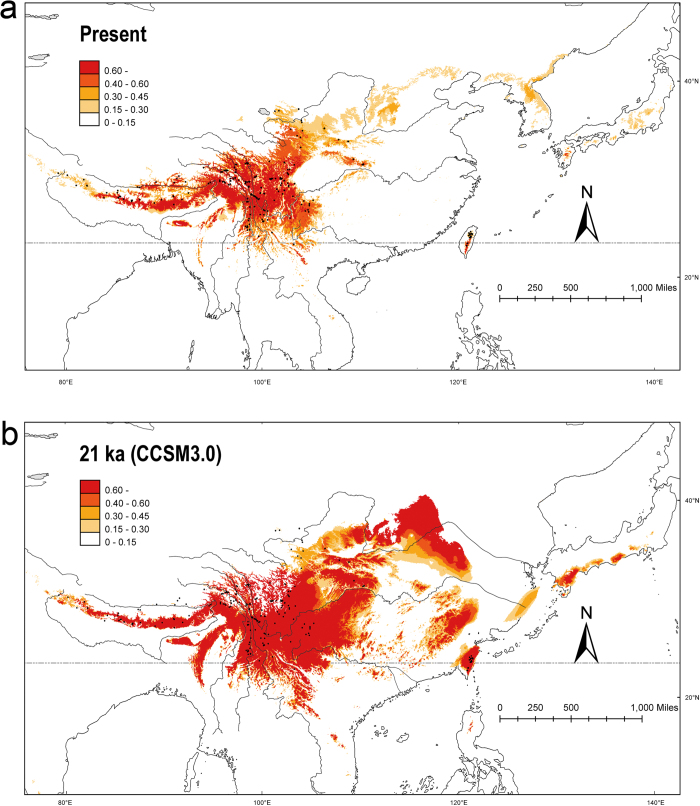
Predicted distribution probability (in logical value) is shown in each 2.5 arc-min pixle, based on the paleodistribution modeling (a) at present (0 BP) and (**b**) at the last glacial maximum (LGM, 21000 BP). Distribution of major river systems on exposed East China Sea during LGM; from Sakaguchi *et al.*^71^. Occurrence records of *R. sericea* complex at present also plotted as black points on maps generated by ESRI ArcGIS 9.3 (ERSI co., USA).

The Maxent model had high predictive power [AUC = 0.986 ± 0.003 (mean ± SD), based on 10 replicates]. The model predicted suitable habitat for the species that was mostly coincident with its natural geographic distribution (Fig. 4a) except that it extended to Korea and Japan, which may not accommodate the species according to specimen records. The main distributional area predicted for the complex was concentrated in southwestern China, mainly at high elevations. To the southeast, the east-west trending Qinling-Dabie-Wuyi mountains showed great possibility for being occupied by the *R. sericea* complex, and this region is located west of Taiwan (Fig. 4b). Obviously, the elevational distribution during the LGM was much lower than at present, and the distribution of *R. sericea* was along the ranges in the east part of Asia which are now occupied by subtropical forests adapted to lower elevations and higher temperatures.

## Discussion

Generally, if deep divergence occurred through paleogeological events, then a phylogeographic study should detect strong geographic clustering that coincides with known geographic boundaries. This scenario appears in some relict plants or relatively old species (e.g., *Cercidiphyllum*^33^). A major finding of the present study was that the *Rosa sericea* complex comprises no distinct geographic lineages as inferred from the chlorotype network and phylogenetic analysis (Figs. 4 and 5), which provided only weak support to divide the complex into three lineages. The three lineages occur throughout the Hengduan Mountains and QTP region (Fig. 3). From the SAMOVA analysis (Fig. S1), the *F*_CT_ increased as more groups were constructed, indicating very weak boundaries among presumed groups based on both geographic and molecular distance (Figs. 1 and 3). Though in nSSR, a weak geographic pattern was detected, however, it was along a continuous gradient from west to east with no distinct boundary (Fig. 2). Based on our results, we suspect physical barriers to past or contemporary dispersal (e.g., the Mekong-Salween divide in Hengduan Mountains region for *Sinopodophyllum hexandrum*^34^; the East China Sea/Tsushima-Korean straits for *Croomia*^35^; the Tokara Gap, a deep sea-barrier in the Ryukyu Islands, for *Neolitsea sericea*^36^) have had limited influence on the distributions of the *R. sericea*. In fact, the relative uniformity among geographic distributions of genotypes suggests sudden demographic expansion, and this type of pattern has been reported recently in *Hippuris vulgaris* (Plantaginaceae)^37^, an aquatic herb distributed on the QTP and in adjacent areas.

*Rosa sericea* emerged in the middle or late Pleistocene (ca. 0.78–0.01 Ma), and its major lineages diversified not earlier than the middle of Pleistocene (Fig. S3). By the time that *R. sericea* evolved, the major geographic features were already well-established. Therefore, the range expansion detected in the *R. sericea* complex is more likely the result of climate oscillations. The relationship between Pleistocene glaciation cycles and demography of organisms is well-documented in the Northern Hemisphere^9^,^10^, including in southern China (reviewed in Qiu *et al.*^6^ and animals^38^,^39^,^40^). Typically, rapid population expansion occurred during interglacial stages and shaped the current distributional patterns and genetic diversity. However, all populations that we sampled demonstrated a sudden radiation 28-15ka (Table 4), which is coincident with Last Glacial Maximum (LGM) of the Pleistocene rather than with an interglacial period. The *R. sericea* complex comprises cold tolerant plants that can survive in the periglacial zone. ENM supports the presence of extensive habitats for the *R. sericea* complex during the LGM (Fig. 4b).

The wide distribution of haplotype H1 and the fact that the expansion hypothesis was not rejected during the late Pleistocene indicate that colonization of most of the QTP and Hengduan Mountains region occurred recently through rapid population expansion (Fig. 1, Table 4). Additionally, the high *F*_ST_ values among populations (*F*_ST_ = 0.822, p < 0.0001, Table 3) indicate significant population diversity. These observations are consistent with the phalanx model of Hewitt^7^, who pointed out that large mountains acting as interglacial refugia may allow many alleles and genomes to survive.

Although the phalanx model may best explain the overall distributional patterns of the *R. sericea* complex, some population features of the group are consistent with the pioneer model. In our case, the pioneer model (edge effect) predicts rapid colonization of an area during glacial periods and fragmentation during interglacials accompanied by genetic drift and range expansions from interglacial refugia over short distances involving limited gene flow during glacial periods^41^. This should result in a clear but weak phylogeographic signature of isolation by distance and detectable interglacial refugia. We observed this in Group IV (chlorotypes H16, H17 and H22) (Figs. 1 and 4). Our genotype data are consistent with several interglacial refugia in populations YDL (H1, H5 and H6) and YLQ (H1, H3, H24 and H32) and in the Hengduan Mountains including SKD, SLD and SXJ (Table 1, Fig. 1). Additionally, range expansion during glacial periods and subsequent range fragmentation probably explains the predominance of the H3 haplotype throughout subtropical area of the Yungui Plateau and the demography of lineage CPA, in which haplotype H1 is predominant (Figs. 1 and 3). Moreover, missing intermediates from the root of H1 (H14, H27, H28 and H30, including H31 as descendant, Fig. 3) are indicative of fragmentation during interglacial periods.

The *Rosa sericea* complex is disjunct between southwest China and Taiwan (Fig. 4). We sampled one population of 15 individuals from Taiwan, and discovered a unique haplotype (H33, Table 1) that is most similar to a population from Xi’an, located in the Qinling Mountains (SHXA) (H18, H21). The Xi’an and Taiwan chlorotypes comprise the CPC lineage. The CPC lineage probably had a widespread distribution from the Qinling-Dabie-Wuyi mountain ranges (Fig. 3) through Taiwan during the LGM when the coastline was much lower (−130 m, ETOPO1) and a land connection existed between Taiwan and mainland China. Our ENM results support the presence of suitable, contiguous habitat for the complex extending from China to Taiwan. An alternative possibility is that the Taiwan lineage represents pre-Pleistocene vicariance associated with the formation of Taiwan island, which may have occurred 10-5 Ma^42^. However, our molecular divergence time estimates show that the CPC lineage diverged from its sister group ca. 0.4–0.7 Ma even when we applied the slowest, most conservative substitution rates (Fig. S3). Thus we reject the possibility of pre-Pleistocene divergence of lineage CPC, and conclude that the colonization of Taiwan is the result of Pleistocene climate oscillation. This finding supports a west to east migration route from southwest China to Taiwan through the Qinling-Dabie Mountains that was first proposed by

Hu^43^,^44^ and clarified by Wang^45^. To further understand this, nuclear SSR cluster analysis using STRUCTURE resolved the Taiwan population in green (cluster 2), which predominated in Hengduan Mountains region indicated a fact that the resident of Taiwan island should be a result of once widely spread *R. sericea* during the past (Fig. 2).

We attempted to use our phylogenetic and population genetic data to delimit species within the *R. sericea* complex. However, our phylogeny showed weakly supported clades and our network analysis resulted in a star-like pattern. Our results also showed low levels of variation among putative species (PV = 7.36%, *F*_CT_ = 0.074, P < 0.05) (Table S4), compared to among population variation within such species (PV = 70.61%, *F*_SC_ = 0.762, P < 0.0001) (Table S4). It is clear that the three clusters identified in present are not associated with the taxonomic identities. Our findings support the continuous variation that we observed in the field and in specimens in herbaria. We propose that the putative species should be treated as *R. sericea*.

In summary, our results show that the cold-tolerant *R. sericea* complex is comprised of a single species that underwent rapid geographic radiation during the LGM. The phylogeographic structure of modern populations is consistent with a phalanx model-type expansion during the LGM and subsequent pioneer model-type population histories. Our work supports the recognition of a single *R. sericea* species.

## Methods

### Plant sampling

Samples were collected from 763 individuals from 62 populations throughout the distribution range of the *Rosa sericea* complex during 2010–2011 (average 11.1 collections per population Fig. 1, Table S1). Leaves were collected and dried by silica gel in the wild, then stored at −80 °C until used. For each population, we sampled 10 or more individuals except that in several sites, because of the rarity of the species, less than 10 individuals were collected. However, in such cases most populations had more the 7 individuals (Table 1). Vouchers were deposited in the Herbarium of the Chengdu Institute of Biology, Chinese Academy of Sciences (CDBI).

### DNA extraction, sequencing, and microsatellite genotyping

Total genomic DNA was extracted from the dried leaf tissue by a modified cetyltrimethyl ammonium bromide (CTAB) method^46^. In a preliminary cpDNA screen, twelve intergenic spacer (IGS) regions were surveyed in ten individuals of the *R. sericea* complex (data not show). Based on the results of amplify efficiency and amount of polymorphism, three IGS, *ndh*J*-trn*F^47^, *trn*L-*trn*F^48^ and *ndh*F-*rpl*32^47^, were adopted for further analysis. PCRs were performed in 25 μL of ca. 50 ng of template DNA, 2.5 μL of 10 × PCR buffer, 2 μL of 2.5 mM dNTPs,0.5 μL of 10 μM each primer and 0.5 unit of TaKaRa ExTaq (TaKaRa). The PCR cycle for all three fragments was as follows: template denaturation at 94 °C for 3 min, followed by 35 cycles of denaturation at 94 °C for 1 min, annealing at 55 °C for 1 min and extension at 72 °C for 1.5 min; followed by a final extension of 72 °C for 10 min. Purified PCR products were sequenced by standard methods on an ABI PRISM 3730 (Applied Biosystems), using the same primers as those of the initial PCR. Forward and reverse sequences were edited manually and aligned by SeqMan Pro 7.1 (implement in DNASTAR, Lasergene). CLUSTALX was used to carry out sequence alignment^49^. All chlorotypes from *Rosa sericea* were deposited in GenBank under accession numbers KF850715 – KF850751, KF850756 – KF850792, KF851050 – KF851086, and for three chloroplast sequences for four outgroup accessions, i.e. *R. hugonis* (2 accessions), *R. banksiae* and *R. cymosa* (KF850752 – KF850755, KF850793 – KF850796, KF851087 – KF851090).

All DNA samples were genotyped at eight nSSR loci using primers and amplification protocols developed for *Rosa*^50^ (see Table S2). A pre-experiment was conducted using twenty pairs of EST primers, and based on the results these eight pairs showed significant polymorphism were chosen in present. PCR products were separated on a MegaBACE 1000 (GE Healthcare Biosciences, Sunnyvale, California, USA). Alleles were scored manually using GENETIC PROFILER (version 2.2; GE Healthcare Biosciences).

### Population genetic and phylogeographic data analyses

To verify the existence of significant heterogeneity in the phylogenetic signal among the three cpDNA fragments, we carried out a partition-homogeneity test^51^ using the software PAUP* 4.0b. As no significant differences were detected, we used the combined cpDNA data set for all subsequent analyses. Indels were reduced and treated as single mutation events and coded as substitutions (A or T).

Sequence variation was analyzed using MEGA 4, whereas nucleotide (*π*) and haplotype (*h*) diversity^52^ was computed using the software DnaSP 5. We also compared population differentiation for phylogenetically ordered (*N*_ST_) and unordered (*G*_ST_) chlorotypes, both in the overall cpDNA data set and within population groups, using PERMUT1.0. The result of this program and the significant test by 1000 permutation were then employed to examine if *N*_ST_ > *G*_ST_, which is a test for the presence of phylogeographic structure. The phylogenetic relationships among chlorotypes were investigated using the statistical parsimony procedure for phylogenetic network estimations implemented in TCS 1.21, with a 95% criterion for a parsimonious connection.

To identify population groups and genetic barriers, a spatial analysis of molecular variance (SAMOVA) was performed using SAMOVA 1.0. Based on a simulated annealing procedure, the SAMOVA algorithm iteratively seeks the composition of a user-defined number of groups (K) of geographically adjacent populations that maximizes the proportion of total genetic variance (*F*_CT_) as a result of differences between groups of populations. We set the number of initial condition to 100 with K = 2–20. To verify its consistency, we ran the analysis five times for each K value with 1000 independent annealing processes. Afterwards, analyses of the molecular variance (AMOVA)^53^ were carried out with the software ARLEQUIN 3.11, using available groupings suggested by SAMOVA and the best-fit models of sequence evolution as indicated by jModelTest. If SAMOVA did not provide suitable barriers, we subdivided groups by geography following Wu *et al.*^54^ with the aim to verify if these traditional boundaries contributed to lineage diversity in cold tolerant groups. Meanwhile, AMOVA was also carried out using subdivided groups based on taxa identified according to treatments in the *Flora of China*^30^ to check whether gene barriers exist among morphologically distinct individuals.

For nSSRs, measures of genetic diversity were assessed for each population without missing data, and across all loci, by calculating the total number of detected alleles (NA), allelic richness (RS), and gene diversity (HS). Differentiation between populations (individuals with missing data were excluded) was computed using FST^55^, and its significance (at each locus, and overall) was tested using 1000 bootstrap permutations. All these analyses were performed in GenALEx (version 6.501) and GENEPOP version 4.0. To characterize population structure, STRUCTURE version 2.3 was run on the entire nSSR dataset (i.e. 62 populations) for 100 000 Markov chain Monte Carlo (MCMC) cycles following 10 000 burn-in cycles, using the admixture model with independent allele frequencies. Ten replications were performed for each K, in the range K = 1–20, and the optimal K was estimated according to Evanno *et al.*^56^.

### Phylogenetic relationship and Divergence time estimates

The phylogeny reconstruction based on haplotype was performed in both Neighbor-joining (NJ) and Bayesian inference (BI) using four accessions of three species, *R. hugonis*, *R. cymosa* and *R. banksiae*, as outgroups based on the framework of *Rosa* phylogeny^29^. The NJ analysis was conducted with MEGA version 4.0, incorporating Kimura’s 2-parameter model of DNA evolution^57^. To evaluate clades support, 1000 bootstrap replicates^58^ were performed using fast heuristic search and tree bisection-reconnection (TBR) branch swapping. BI analysis was conducted using MrBayes version 3.1.2. The best substitution model for BI analysis was identified using the Akaike Information Criterion (AIC)^59^, which is determined via jModelTest version 2.1.4. The Markov chain Monte Carlo (MCMC) algorithm was run for 50,000,000 generations, with four incrementally heated chains. The analysis involved starting from a random tree and sampling every 1000 generations. The first 20% of trees were treated as burn-in, and discarded. The remaining trees were used to construct a Bayesian consensus tree.

Using the same matrix as above, tree topologies and node ages were determined in BEAST version 1.61 using a GTR+I substitution model, selected by jModelTest, and an uncorrelated lognormal relaxed clock^60^. A Yule process was specified as tree prior. Although there are fossil records of *Rosa*^61^, the absolutely position or the nearest living relatives of these fossils are not easy to determine. Since, using remote calibrations might also not accurately determine terminal members, we adopted a substitution rate method. A likelihood ratio test in PAUP 4.10b suggested that the chloroplast dataset rejected a strict molecular clock (P < 0.01, data not shown), and therefore we used relaxed molecular clock approaches. In this study, as the lineage-specific cpDNA substitution rate for *Rosa sericea* is unknown, three different cpDNA substitution rates for angiosperms [the slowest (1.1 × 10^−9^ substitution/site/year), fastest (2.9 × 10^−9^ s/s/y) and mean (2.0 × 10^−9^ s/s/y)^62^,^63^ ]were employed to estimate divergence times. We ran two independent Markov chains for 50,000,000 generations each from a random starting tree. The chains were sampled every 1,000 generations, but the first 20% of these were eliminated as burn-in. All log and tree files from independent, simultaneous runs were combined using LogCombiner, and maximum credibility trees were generated in TreeAnnotator using the product method^64^.

### Demographic analysis

For a single expanding subclade identified (see the Results section), demographic history was initially explored using mismatch distribution analysis (MDA), which represented the frequency distribution of pair-wise nucleotide differences among all chlorotypes in the population(s)^31^. A population in demographic equilibrium is characterized by multimodal mismatch distributions, whereas populations that have experienced recent demographic expansion should show smooth unimodal distributions. The distribution of the number of pairwise differences between chlorotypes with their theoretical distribution expected under a model of sudden (stepwise) demographic expansion. Goodness of fit was tested with the sum of squared deviations (*SSD*) between observed and expected mismatch distributions and Harpending’s raggedness index (*H*_Rag_)^65^ using 1000 bootstrap replicates. We conducted this analysis in ARLEQUIN 3.11^66^ to test for demographic patterns in the major regional groups identified by phylogenetic analysis of TCS (see the Results section).

In such a context, two methods were used to estimate the time of expansion of the *Rosa sericea* complex. First, the expansion time was estimated directly from the mismatch distribution with the statistic *τ* (tau) and translated into absolute time in years (t), using the equation *T* = τ/2*u* (*T*, in number of generations), where *u* is the neutral mutation rate for the entire sequence per generation and is calculated as *u* = *μkg*, where *μ* is the substitution rate in substitutions per site yr^−1^ (s/s/y), *k* is the average sequence length of the cpDNA region under study (k = 2974 in this study), and *g* is the generation time in yr (i.e. age of first reproduction; approximated as 3 yr, as observed for *Rosa* cultivation by X. F. Gao). The substitution rate range of the three combined cpDNA-IGS regions was assumed as minimum and maximum mutation rates of 1.1 × 10^−9^ s/s/y and 2.9 × 10^−9^ s/s/y^62^,^63^. To obtain estimates of changes in demographic growth over the history of major areas, the historical demographic dynamics of the *R. sericea* complex were inferred from Bayesian skyline plot (BSP) analyses using BEAST 1.6.1^64^. The BSP analyses are preferred because multiple loci are used to estimate effective population size through time. Linear and stepwise models were explored using an uncorrelated lognormal relaxed clock. Runs consisted of 50,000,000 generations, with trees sampled every 1000 generations. The BSP was visualized in the program Tracer version 1.4, which summarizes the posterior distribution of population size over time. In addition, we used Tajima’s *D* test^67^ and Fu’s *F*_S_ test^68^ as implemented in ARLEQUIN version 3.1.1 to detect evidence of a recent demographic expansion within each inferred biogeographical region. The significance of deviation from value was tested with 10,000 bootstrap replicates.

### Present and past distribution modeling

To reconstruct the past distribution of the *R. sericea* complex during cold periods, species distribution models for the *R. sericea* complex were generated using MAXENT 3.3.3k. MAXENT is a program for maximum entropy modeling of the geographical distributions of species; it combines presence only data with ecological climatic layers to predict species occurrence in areas where data points are unavailable. We used MAXENT to predict species occurrence under both present day and LGM conditions. Although potentially useful for this study, we avoided making predictions for the last interglacial phase (about 135 000–115 000BP), because of a lack of detailed bioclimatic data for this period. Information on the geographic distribution of the *R. sericea* complex was based on present collection and herbarium specimens from the herbaria A, BM, CDBI, E, HNWP, IBSC, K, KATH, KUN, PE, TUCH and WUK. In total, 317 presence records of members of the *R. sericea* complex were obtained from throughout the species range, after removal of duplicate records within each pixel (2.5 arc-min; ~5 km). Six bioclimatic variables (Hijmans *et al.* 2005) at 2.5 arc-min resolution were used to model the species’ niche: (i) annual mean temperature, (ii) mean temperature of the warmest quarter, (iii) mean temperature of the coldest quarter, (iv) annual precipitation, (v) precipitation of the wettest quarter and (vi) precipitation of the driest quarter. Model validation was performed using default settings with 100 replicates of cross-validation procedures with 25% of the data used for model testing. The area under the ROC curve (AUC) was then calculated for each run, which is an indicator of the accuracy of the model prediction^69^.

The established model was then projected onto the reconstructed climatic conditions during the LGM simulated by Community Climate System Model 3.0^70^, downloaded from the WorldClim database website (http://www.worldclim.org). LGM paleo climate layers in 2.5 arc-min resolution were then prepared from these data following Sakaguchi *et al.*^71^. To estimate the paleo coastlines (−130 m than at present) and the paleo climate surfaces of the exposed seafloor area during the LGM, seafloor topography data (ETOPO1 bedrock) were downloaded from the National Geophysical Data Center of the National Oceanic and Atmospheric Administration (NOAA, USA) and used to adjust the coastline of LGM in ArcGIS 9.3 (ERSI co., USA).

## Additional Information

**How to cite this article**: Gao, Y.-D. *et al.* Pleistocene glaciations, demographic expansion and subsequent isolation promoted morphological heterogeneity: A phylogeographic study of the alpine *Rosa sericea* complex (Rosaceae). *Sci. Rep.* **5**, 11698; doi: 10.1038/srep11698 (2015).

## Supplementary Material

Supplementary Figures 1-4

Supplementary Table S1

Supplementary Table S2

Supplementary Table S3

Supplementary Table S4

## Figures and Tables

**Table 1:** Detected chlorotypes and the genetic diversity identified in this study.

Population code	Taxon code*	Regional group	Line age	Sample size	Haplo types (no. of individuals)	Hd (SD)	π (SD)
		I: Qinghai–Xizang Plateau region				0.799(0.020)	0.00078(0.00007)
XAR	RS, RO	I	A	13	H1 (4), H28 (9)	0.462(0.110)	0.00062(0.00015)
XBR	RO	I	A	13	H1 (1), H15 (12)	0.154(0.126)	0.00005(0.00004)
XCN	RO	I	A	8	H30 (8)	0.000	0.00000
XDJ	RO	I	A	8	H31 (8)	0.000	0.00000
XMZ	RO	I	A	6	H1 (4), H15 (1), H29 (1)	0.600(0.215)	0.00022(0.00009)
XJL	RO	I	A	11	H1 (11)	0.000	0.00000
XNML	RS, RO	I	A	8	H1 (4), H29 (4)	0.571(0.094)	0.00019(0.00003)
XYD	RO	I	A	12	H30 (12)	0.000	0.00000
		II: Eastern Himalayas & Hengduan Mountains region				0.579(0.031)	0.00043(0.00004)
SBT	RO	II	A	6	H1 (6)	0.000	0.00000
SDC1	RO	II	A	16	H1 (15), H24 (1)	0.125(0.106)	0.00025(0.00021)
SDC2	RM	II	A	20	H1 (20)	0.000	0.00000
SDC3	RK	II	A	8	H1 (8)	0.000	0.00000
SEM	RO	II	A	13	H1 (11), H33 (2)	0.282(0.142)	0.00009(0.00005)
SKD1	RS	II	B	10	H7 (1), H8 (7), H9 (2)	0.511(0.164)	0.00038(0.00012)
SKD2	RO	II	B	10	H1 (1), H3 (9)	0.2(0.154)	0.00007(0.00005)
SLD1	RO	II	B	10	H9 (6), H11 (4)	0.533(0.095)	0.00018(0.00003)
SLD2	RS	II	B	6	H23 (6)	0.000	0.00000
SXC1	RM	II	A	9	H1 (7), H25 (2)	0.389(0.164)	0.00013(0.00006)
SXC2	RK	II	A	17	H1 (12), H25 (5)	0.441(0.098)	0.00015(0.00003)
SXJ1	RO	II	B	10	H36 (8), H37 (2)	0.356(0.159)	0.00024(0.00011)
SXJ2	RO	II	B	15	H3 (15)	0.000	0.00000
SYJ1	RS, RO	II	A&C	18	H1 (16), H10 (2)	0.209(0.116)	0.00007(0.00004)
SYJ2	RO	II	A&C	18	H1 (17), H10 (1)	0.111(0.096)	0.00004(0.00003)
XBM1	RO	II	A	10	H1 (8), H13 (2)	0.356(0.159)	0.00012(0.00005)
XBM2	RO	II	A	7	H1 (6), H14(1)	0.286(0.196)	0.00019(0.00013)
XBS1	RK	II	A	13	H1 (7), H13 (6)	0.538(0.060)	0.00018(0.00002)
XBS2	RO	II	A	7	H1 (7)	0.000	0.00000
XBS3	RK	II	A	10	H1 (9), H13 (1)	0.200(0.154)	0.00007(0.00005)
XCY	RO	II	A	7	H1 (3), H12 (3), H13 (1)	0.286(0.196)	0.0001(0.00007)
XJD	RO	II	A	13	H1 (11), H16 (2)	0.282(0.142)	0.00009(0.00005)
XLL1	RK	II	A	7	H1 (6), H15(1)	0.286(0.196)	0.0001(0.00007)
XLL2	RS	II	A	7	H1 (7)	0.000	0.00000
XLZ1	RO	II	A	16	H1 (1), H14 (5), H27 (12)	0.542(0.098)	0.00042(0.00010)
XLZ2	RM	II	A	16	H1 (14), H15 (1), H28 (1)	0.242(0.135)	0.00021(0.00015)
XMK1	RO	II	A	10	H1 (10)	0.000	0.00000
XMK2	RS	II	A	9	H1 (6), H12 (3)	0.5(0.128)	0.00084(0.00022)
XMK3	RK	II	A	9	H1 (8), H12 (1)	0.222(0.166)	0.00037(0.00028)
YFG	RO, RT	II	A	15	H1 (8), H2 (7)	0.533(0.052)	0.00018(0.00002)
YZD1	RO	II	A	10	H1 (2), H26 (8)	0.356(0.159)	0.00024(0.00011)
		III: Yungui Plateau region				0.558(0.044)	0.00045(0.00004)
GUWN	RS	III	B	12	H3 (10), H19 (2)	0.303(0.147)	0.0001(0.00005)
SJL	RM	III	B	10	H3 (10)	0.000	0.00000
SMG	RS	III	B	13	H3 (13)	0.000	0.00000
SMN1	RS	III	B	9	H3 (6), H4 (3)	0.500(0.128)	0.00017(0.00004)
SMN2	RS, RO	III	B	10	H3 (10)	0.000	0.00000
YDL	RO	III	B	8	H1 (3), H5 (2), H6 (3)	0.536(0.123)	0.00018(0.00004)
YJD	RS	III	B	12	H6 (12)	0.000	0.00000
YLQ	RS	III	B	8	H1(1), H3 (2), H24 (1), H32 (4)	0.714(0.123)	0.00034(0.00007)
YTC	RM	III	B	14	H3 (14)	0.000	0.00000
YWX	RS	III	B	11	H3 (9), H19 (2)	0.327(0.153)	0.00011(0.00005)
YYD	RO	III	B	11	H6 (1), H35 (10)	0.182(0.144)	0.00006(0.00005)
YZD2	RO, RZ	III	B	7	H3 (7)	0.000	0.00000
		IV: Qilian-Qin Mountains region				0.598(0.0051)	0.00047(0.00006)
GHZ	RO	IV	A	8	H14 (5), H15 (3)	0.536(0.123)	0.00018(0.00004)
GJT	RO	IV	A	14	H17 (9), H22 (5)	0.495(0.088)	0.00017(0.00003)
GYZ	RO	IV	A	11	H16 (7), H17 (4)	0.509(0.101)	0.00017(0.00003)
GZL	RO	IV	A	12	H17 (12)	0.000	0.00000
HXS	RO	II	A	17	H1 (6), H20 (11)	0.485(0.079)	0.00016(0.00003)
QDT	RO	IV	A	13	H17 (13)	0.000	0.00000
SHPL	RO	II	A	7	H1 (7)	0.000	0.00000
SHXA	RO	IV	C	12	H18 (8), H21 (4)	0.485(0.106)	0.00016(0.00004)
SJZ	RO	IV	A	12	H6 (1), H7 (8), H17 (3)	0.530(0.136)	0.0003(0.00008)
SSD	RO	II	A	14	H1 (2), H16 (4), H17 (7), H18 (1)	0.538(0.115)	0.00029(0.00012)
		V: Taiwan					
THL	MO	V	C	15	H33 (15)	0.000	0.00000
Total						0.815(0.017)	0.00056(0.00003)

^*^RO = *Rosa omeiensis*, RS = *R. sericea*, MO = *R. morrisonensis*, RK = *R. sikangensis*, RT = *R. taronensis*, RM = *R. mairei*, RZ = *R. zhongdianensis*.

**Table 2:** Genetic diversity parameters for all 62 populations of *Rosa sericea* complex and for four group separately.

	*H*_S_	*H*_T_	*N*_ST_	*G*_ST_	permutation test (P)
All	0.267 (0.0309)	0.823 (0.0352)	0.766 (0.0367)	0.676 (0.0371)	*N*_ST_ > *G*_ST_ (*P *< 0.01)
Group I	0.223 (0.0967)	0.872 (0.0464)	0.834 (0.0672)	0.744 (0.1170)	*N*_ST_ > *G*_ST_ (*P *> 0.05)
Group II	0.265 (0.0375)	0.599 (0.0823)	0.746 (0.0429)	0.558 (0.0651)	*N*_ST_ > *G*_ST_ (*P *< 0.01)
Group III	0.264 (0.0824)	0.707 (0.1221)	0.720 (0.0725)	0.627 (0.0988)	*N*_ST_ > *G*_ST_ (*P *> 0.05)
Group IV	0.373(0.0836)	0.814(0.0768)	0.746 (0.0813)	0.541 (0.0989)	*N*_ST_ > *G*_ST_ (*P *< 0.05)

*H*_S_, average genetic diversity within populations; *H*_T_, total genetic diversity; *N*_ST_, inter-population differentiation taking into account sequence difference; *G*_ST_, inter-population differentiation.

**Table 3:** Hierarchical analysis of molecular variation (AMOVA) based on data from the three chloroplast DNA spacers for all 62 populations of *Rosa sericea* complex and for five groups separated by geographic features.

Regional grouping of populations	Source of variation	d.f.	SS	VC	PV	*F*- statistics
All	Among groups	4	160.148	0.280	25.33	*F*CT = 0.253**
	Among populations within groups	57	405.876	0.627	56.82	*F*SC = 0.761**
	Within populations	629	123.909	0.197	17.85	*F*ST = 0.822**
Group I	Among populations	7	75.118	1.074	82.96	*F*ST = 0.830**
	Within populations	71	15.667	0.221	17.04	
Group II	Among populations	30	190.115	0.554	73.16	*F*ST = 0.732**
	Within populations	321	63.93	0.199	26.84	
Group III	Among populations	11	72.735	0.616	74.10	*F*ST = 0.741**
	Within populations	113	24.337	0.215	25.90	
Group IV	Among populations	9	82.533	0.746	73.66	*F*ST = 0.737**
	Within populations	110	29.35	0.267	26.34	
Group V	Among populations	/	/	/	/	
	Within populations	/	/	/	/	
Total populations	Among populations	61	591.005	0.848	77.33	*F*ST = 0.773**
	Within populations	629	156.384	0.249	22.67	

d.f., degree of freedom; SS, sum of squares; VC, variance components; PV, percentage of variation

**P < 0.0001

**Table 4:** Mismatch distribution analysis (MDA) of cpDNA sequence data for *Rosa sericea* complex from lineages as a whole and three lineages recognized by phylogenetic inferences.

Line ages	Tajima’s D (p value)	Fu’s FS (p value)	τ	tmin (Ma)	tmax(Ma)	MDA	SSD (p value)	HRag (p value)
Over all	−1.6323(0.0140)	−20.9098(0.0000)	0.734(0.609–1.125)	0.04255(0.03531–0.06522)	0.11218(0.09309–0.17194)	Unimodal	0.1185(0.0000)	0.0234(1.0000)
CPA	−1.5871(0.0250)	−6.4842(0.0440)	1.052(0.000–3.436)	0.06099(0.00000–0.19920)	0.16079(0.00000–0.52516)	Unimodal	0.0009(0.8740)	0.0322(0.9390)
CPB	0.1675 (0.6150)	−1.8907(0.2550)	4.328(0.139–8.531)	NC	NC	Multimodal	0.0173(0.5390)	0.0637(0.6130)
CPC	1.6769(0.9410)	2.7972(0.8860)	3.317(0.000–10.373)	NC	NC	Bimodal	0.0855(0.0540)	0.2293(0.0090)

The values of the neutrality tests for each lineage were examined by Tajima’s D and Fu’s *Fs* statistics. Goodness of fit of observed-to-theoretical mismatch distributions under a sudden (stepwise) expansion model (Rogers and Harpending 1992) is test with the sum of squared deviations (*SSD*) and Harpending’s raggedness index (*H*_Rag_). Upper and lower 95% confidence limits around estimates of *τ* and associated ranges of *t* (in million years before present, Ma) are in parentheses.

NC, Not calculate
