# Supplementary material for: Pleistocene glaciations, demographic expansion and subsequent isolation promoted morphological heterogeneity: A phylogeographic study of the alpine Rosa sericea complex (Rosaceae)
Source: Sci Rep. 2015 Jun 30;5:11698. doi: 10.1038/srep11698 (PMC5155592; doi:10.1038/srep11698)
Supplement: Supplementary Figures 1-4 [file srep11698-s1.pdf]

Pleistocene glaciations, demographic expansion and subsequent isolation promoted morphological heterogeneity: A phylogeographic study of the alpine *Rosa sericea* complex (Rosaceae)

Yun-Dong Gao[1], Yu Zhang[1], Xin-Fen Gao[1,2], Zhang-Ming Zhu[1]

1 Key Laboratory of Mountain Ecological Restoration and Bioresource Utilization & Ecological Restoration Biodiversity Conservation Key Laboratory of Sichuan Province, Chengdu Institute of Biology, Chinese Academy of Sciences

2 Author for correspondence, email: xfgao@cib.ac.cn

Supplementary info

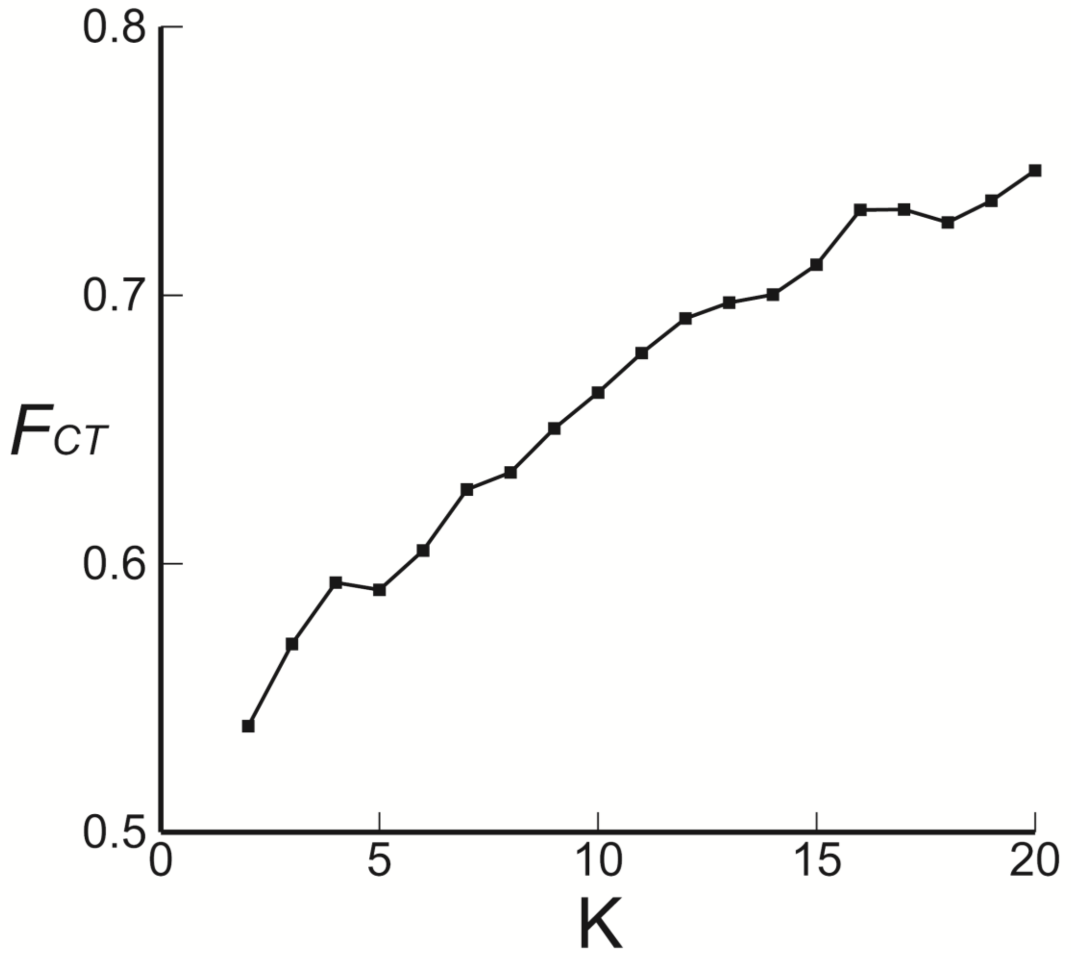

Figure S1. Correlation between the number of groups (*K*) and genetic variance (F_{CT}) in SAMOVA analysis.

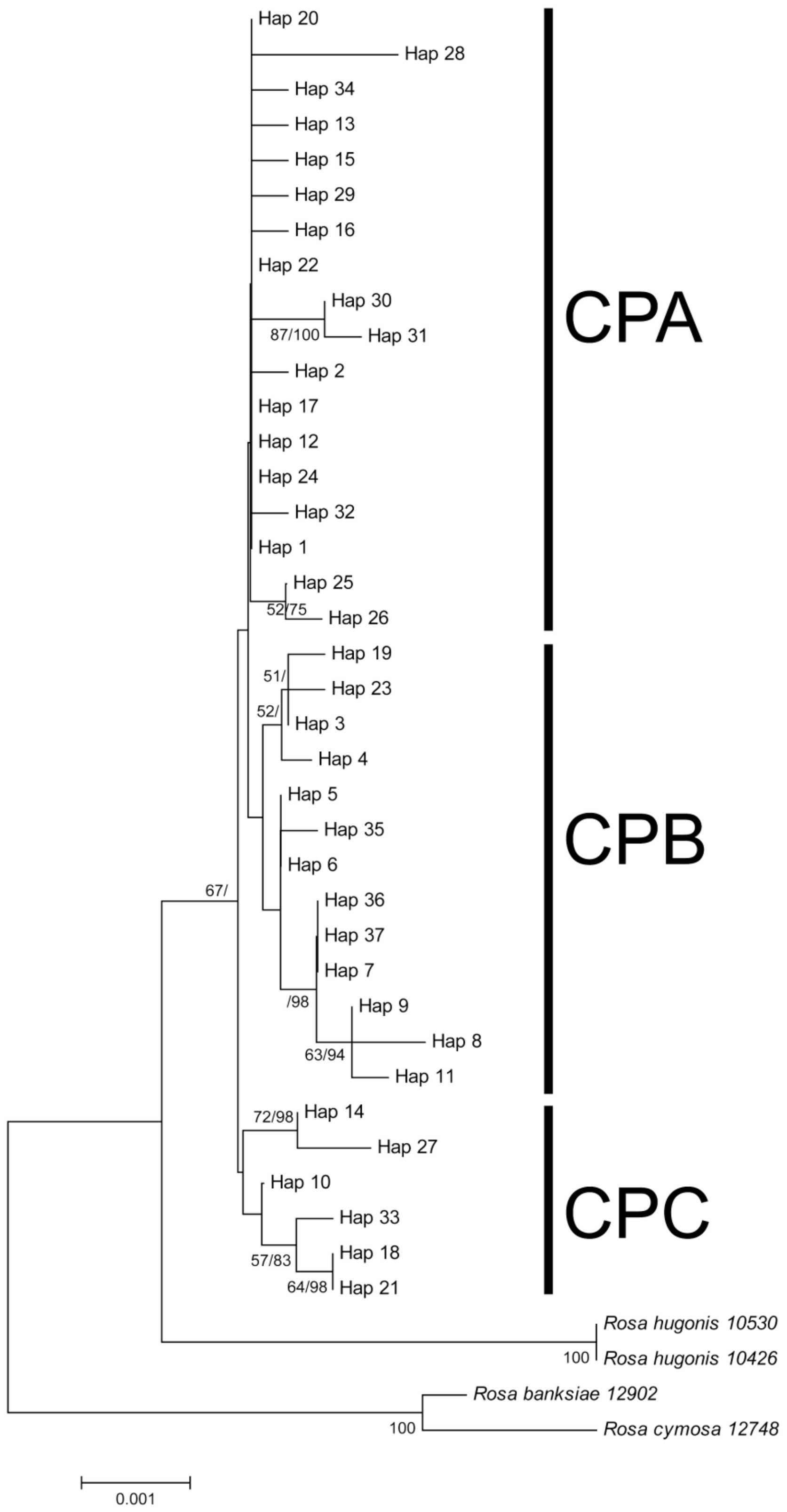

Figure S2. Phylogenetic topography based on plastid DNA dataset. Bootstrap values (BP) of Neighbor-joining analysis and posterior probabilities of Bayesian inferences (PP) are given as BP/PP format. Lineages were detected and labeled as CPA – CPC, respectively.

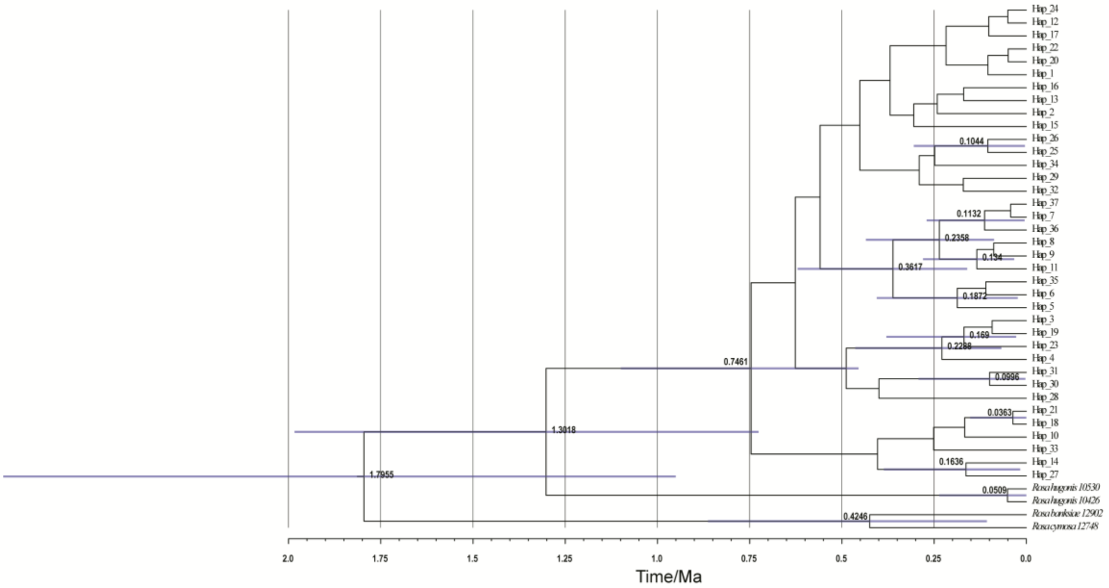

Figure S3. BEAST-generated maximum clade credibility tree of 37 haplotypes (H1-H37) identified in *Rosa sericea* complex, average substitution rate of 2.0×10^{-9} s⁄s⁄y was adopted.

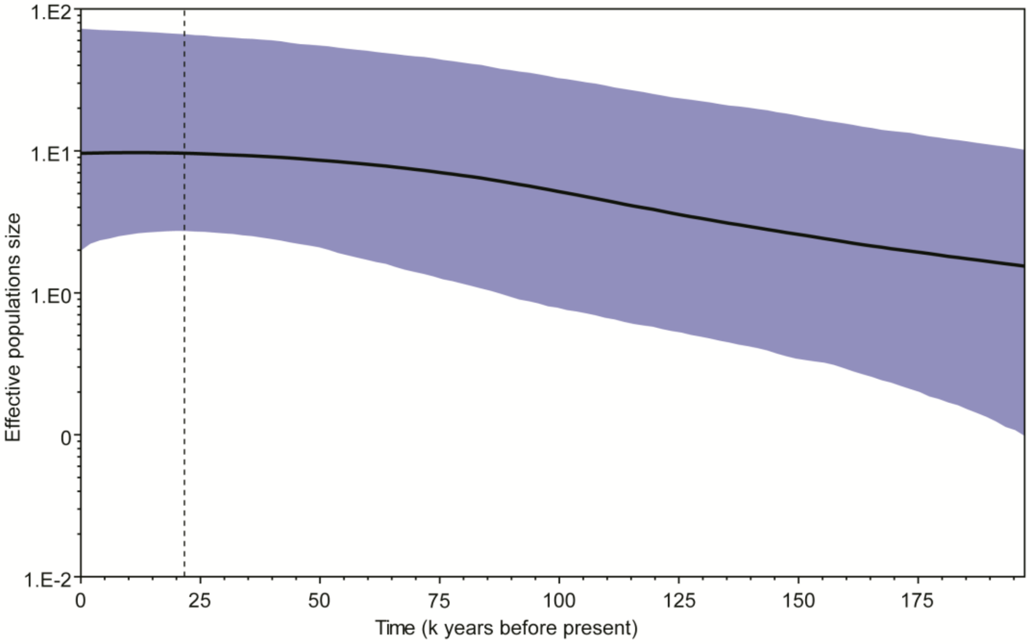

Figure S4. Bayesian skyline spot (BSP) estimate by BEAST for the whole populations of *Rosa sericea* complex: x-aixs, time in kilo of years; y-axis, effective population size. The mean estimate and both 95% HPD limits are indicated. Dash line indicate the Last Glacial Maximum (LGM).
