# Supplementary material for: Pleistocene glaciations, demographic expansion and subsequent isolation promoted morphological heterogeneity: A phylogeographic study of the alpine Rosa sericea complex (Rosaceae)
Source: Sci Rep. 2015 Jun 30;5:11698. doi: 10.1038/srep11698 (PMC5155592; doi:10.1038/srep11698)
Supplement: Supplementary Table S2 [file srep11698-s3.doc]

**Table S2** Mean estimates of genetic diversity and differentiation at eight nuclear microsatellite loci surveyed across all populations

| Locus | *N*_A_ | *H*_O_ | *H*_S_ | *H*_T_ | *F*_ST_ |
| --- | --- | --- | --- | --- | --- |
| EC587106 | 13 | 0.364 | 0.392 | 0.703 | 0.442 |
| EC586919 | 23 | 0.252 | 0.400 | 0.698 | 0.426 |
| BQ106227 | 18 | 0.333 | 0.501 | 0.763 | 0.344 |
| EC587073 | 22 | 0.500 | 0.599 | 0.706 | 0.151 |
| CF349346 | 36 | 0.481 | 0.723 | 0.921 | 0.215 |
| EC587962 | 22 | 0.584 | 0.629 | 0.855 | 0.264 |
| EC587071 | 32 | 0.671 | 0.725 | 0.910 | 0.203 |
| EC587517 | 29 | 0.534 | 0.565 | 0.902 | 0.374 |
| mean | 24.4 | 0.465 | 0.567 | 0.807 | 0.298 |

*N*_A_, observed allele number; *H*_O_, observed within-population heterozygosity; *H*_S_, expected within-population (gene) diversity; *H*_T_, overall gene diversity; *F*_ST_, among-population differentiation.
